# Molecular Detection, Serotyping, and Antibiotic Resistance of Shiga Toxigenic *Escherichia coli* Isolated from She-Camels and In-Contact Humans in Egypt

**DOI:** 10.3390/antibiotics10081021

**Published:** 2021-08-23

**Authors:** Mohamed Said Diab, Reda Tarabees, Yasser F. Elnaker, Ghada A. Hadad, Marwa A. Saad, Salah A. Galbat, Sarah Albogami, Aziza M. Hassan, Mahmoud A. O. Dawood, Sabah Ibrahim Shaaban

**Affiliations:** 1 Department of Animal Hygiene and Zoonoses, Faculty of Veterinary Medicine, New Valley University, El Kharga 72511, Egypt; mohameddiab333@gmail.com; 2 Department of Bacteriology, Mycology, and Immunology, Faculty of Veterinary Medicine, University of Sadat City, Sadat City 32897, Egypt; reda.tarabees@vet.usc.edu.eg; 3 Department of Animal Medicine (Infectious Diseases), Faculty of Veterinary Medicine, The New Valley University, El Kharga 72511, Egypt; yasserelnaker@yahoo.com (Y.F.E.); salahgalbat@yahoo.com (S.A.G.); 4 Department of Animal Hygiene and Zoonoses, Faculty of Veterinary Medicine, University of Sadat City, Sadat City 32897, Egypt; ghadavet@yahoo.com; 5 Department of Food Control, Faculty of Veterinary Medicine, Menofia University, Menofia, ShebinAl-Kom 32511, Egypt; Drmarwa2200@gmail.com; 6 Department of Biotechnology, College of Science, Taif University, P.O. Box 11099, Taif 21944, Saudi Arabia; dr.sarah@tu.edu.sa (S.A.); a.hasn@tu.edu.sa (A.M.H.); 7 Department of Animal Production, Faculty of Agriculture, Kafrelsheikh University, Kafrelsheikh 33512, Egypt; 8 The Center for Applied Research on the Environment and Sustainability, The American University in Cairo, Cairo 11835, Egypt; 9 Department of Animal Hygiene and Zoonoses, Faculty of Veterinary Medicine, Damanhour University, Damanhour 22511, Egypt; sabah_ibrahim@vetmed.dmu.edu.eg or

**Keywords:** antibiotic resistance, camel, *Escherichia coli*, domestic, milk, mastitis

## Abstract

This study aims to determine the prevalence of STEC in she-camels suffering from mastitis in semi-arid regions by using traditional culture methods and then confirming it with Serological and molecular techniques in milk samples, camel feces, as well as human stool samples for human contacts. In addition, an antibiotic susceptibility profile for these isolates was investigation. Mastitic milk samples were taken after California Mastitis Test (CMT) procedure, and fecal samples were taken from she-camels and human stool samples, then cultured using traditional methods to isolate *Escherichia* *coli*. These isolates were initially classified serologically, then an mPCR (Multiplex PCR) was used to determine virulence genes. Finally, both camel and human isolates were tested for antibiotic susceptibility. Out of a total of 180 she-camels, 34 (18.9%) were mastitic (8.3% clinical and 10.6% sub-clinical mastitis), where it was higher in camels bred with other animals. The total presence of *E. coli* was 21.9, 13.9, and 33.7% in milk, camel feces, and human stool, respectively, whereas the occurrence of STEC from the total *E. coli* isolates were 36, 16, and 31.4% for milk, camel feces, and stool, respectively. Among the camel isolates, *stx*_1_ was the most frequently detected virulence gene, while *hlyA* was not detected. The most detected virulence gene in human isolates was *stx*_2_ (45.5%), followed by *stx*_1_. Camel STEC showed resistance to Oxytetracycline only, while human *STEC* showed multiple drug resistance to Amoxicillin, Gentamycin, and Clindamycin with 81.8, 72.7, and 63.6%, respectively. Breeding camels in semi-arid areas separately from other animals may reduce the risk of infection with some bacteria, including *E. coli*; in contrast, mixed breeding with other animals contributes a significant risk factor for STEC emergence in camels.

## 1. Introduction

Camels are characterized by their remarkable ability to adapt to the extreme desert ecosystem and their high resistance to many pathogenic microorganisms (MOs) compared to other domesticated animals in the same area [1]. Dromedary camels contribute strongly to human survival in the Middle East, and North and East Africa regions. The main reason for raising camels is to produce milk, as camels produce more milk for a longer period when compared to other dairy animals. However, its role in human transportation and as an essential source of meat cannot be ignored [2,3,4]. Camel milk is one of the main and important components in the human diet in these regions because it has a high nutritious value such as a high proportion of vitamin C, antibacterial substance, lactoferrin as well as some minerals, and minimum sugar and cholesterol content in comparison to cow milk [5,6].

Few published scientific studies are dealing with the causative agents of camel diseases, including mastitis [7], which is one of the most important diseases that affect dairy animals, that results in severe economic losses, including a decrease in milk yield, and the cost of treatment in addition to the public health risks [8,9,10]. Mastitis is an uncommon disease in camels compared to cows, but its incidence often increases with several things, including teat deformities, hand milking, and herd management [6]. On the other hand, Bessalah et al. [11] pointed to camel diarrhea as the main cause of economic loss associated with poor growth, medication costs, and animal death. Mastitis has extreme zoonotic and economic importance since it causes multiple hazardous effects on human health and animal production. Moreover, in these regions, the daily consumption of camel milk mainly occurs in the raw form [12,13].

Shiga Toxigenic *Escherichia coli* (STEC) is a significant foodborne zoonotic pathogen responsible for mild to severe diarrhea, hemorrhagic colitis, and hemolytic uremic syndrome. STEC virulence factors are derived from Shiga toxin genes (*stx*_1_ and *stx*_2_), which are the chief factors accountable for the clinical signs, intimin (*eae*), and hemolysin (*hlyA*) [14,15]. Hand-to-mouth transfer, considered as direct contact with farm animals, is the dominant mode of STEC transmission to human. Ruminants, mainly cattle, are considered the primary source of STEC infection for humans [16,17]; some authors exclude the role of camels [18,19]. Diagnostic methods using molecular techniques are faster and more accurate than traditional culturing methods for determining the different bacterial species [20].

The excessive and misuse of antibiotics in humans, animals, and plants was considered one of the main contributors to increasing the incidence of multidrug-resistant bacteria [21,22,23]. In the past, some authors asserted that there were no multiple drug-resistant bacteria among the causes of mastitis [24,25]. However, recently, bacteria such as *E. coli* have been discovered that are resistant to many antibiotics, which may be transmitted from milk-producing cows to humans [26,27]. Little information is presented about antimicrobial resistance among pathogenic MOs in camel [28]. Even if the resistance rates to antibiotics are relatively low, it can be dangerous due to the possibility of the transmission and spread of resistance genes between strains [29]. The nomadic nature of this region and the reliance on medicinal plants as natural antibacterial agents may have been an influential factor in the discovery of low levels of multi-antibiotic resistance [6,30,31,32]. This may have a positive impact on both veterinary and public health [33]. Consequently, this work aimed to study the role of *STEC* in mastitis and diarrhea in she-camels and its incidence among human beings in the same area. Additionally, antibiotic susceptibility tests for the isolates were performed.

## 2. Results

The data presented in Table 1 revealed that the general occurrence of camel mastitis was 18.9% (8.3% clinical and 10.6% subclinical mastitis). Moreover, the results presented in Table 2 showed that the prevalence of clinical and subclinical mastitis among she-camels reared separately with no contact with other animal species was 4.1 and 5.6%, respectively, while the rates of infection in camels raised with other animal species increased, reaching 11.1 and 13.9%, respectively. There was a statistically significant difference between the two breeding systems, either in separate or mixed breeding.

The results presented in Table 3 showed that *E. coli* was isolated using conventional culture methods from 25.6 and 19.7% of the examined clinically and sub-clinically mastitic camel’s milk. Concerning the isolation of *E. coli* from the fecal samples, our results showed that *E. coli* was isolated from 44.4 and 12.3% of the examined fecal samples collected from diarrheic camels and apparently healthy she-camels, correspondingly, as shown in Table 3.

Regarding human stool samples (Table 4), our results showed that *E. coli* was isolated from 23.2 and 37.2% of the examined stool samples collected from contact and non-contact individuals, respectively, with no statistically significant difference (*p* > 0.05). Among these isolates, STEC represented 16.7 and 34.5%, respectively. Concerning the seasonal prevalence of STEC, our results presented in Table 5 revealed a higher prevalence of STEC in a cold climate than in hot climates.

Table 6 showed that six different STEC serotypes were recovered from camel samples, including O26, O45, O103, O111, O121, and O145 in percentages of 6, 2, 2, 6, 4, and 6%, respectively, whereas O26, O45, O103, and O145 serotypes were recovered from human stool samples in percentages of 11.4, 8.6, 5.7, and 5.7%, respectively.

Table 7 showed that 13 (26%) and 11 (31.4%) of the examined *E. coli* isolates recovered from camel and human samples, respectively, were positive for at least one of the examined genes for *STEC*. Among the tested camel isolates, the most prevalent virulence factors were *stx*_1_, *Eae*, and *stx*_2_ by rates of 46.2, 30.7, and 23.1%, respectively.

In the present study, 13 and 11 of the STEC isolates recovered from camel and human samples, respectively, were screened for their antimicrobial susceptibility, as shown in Table 8. The current study results showed that the camel STEC isolates were sensitive to the most tested antibiotics, except for Oxytetracycline, to which the isolates showed resistance with 53.8%, while the human isolates of STEC showed the highest resistance to Amoxicillin, Gentamycin, and Clindamycin with ratios of 81.8, 72.7, and 63.6, respectively.

## 3. Discussion

There is a dearth of information on STEC epidemiology in humans, food, and animals in Sub-Saharan Africa, and the current knowledge of STEC sources needs to be further improved [17]. Similarly, there is limited information on the occurrence and the characteristics of *STEC* in African camels. Therefore, the current study was undertaken to estimate STEC incidence in the mastitic milk and fecal samples of dromedary camels and in-contact human stool. In addition, the isolates were further characterized for the presence of some virulence encoding genes and antibiogram sensitivity patterns.

In the present study, a total of 180 she-camels were investigated for the presence of clinical and sub-clinical mastitis; the results revealed that the general occurrence of camel mastitis was 18.9% (8.3% clinical and 10.6% subclinical mastitis). However, the general occurrence on the udder quarters level was 6.9%. These results were nearly similar to Jilo et al. [6], who stated that subclinical mastitis was more prevalent than clinical mastitis. Higher results, 26.3%, were reported by Balemi et al. [35]. Moreover, the results showed that clinical and subclinical mastitis prevalence among she-camels reared separately with no contact with other animal species was 4.1 and 5.6%, respectively, while the infection rates in camels reared with other animals increased, reaching 11.1 and 13.9%, respectively. There was a statistically significant difference between the two breeding systems, either in separate or mixed breeding. Similar findings were validated by Clement et al. [23] due to the possibility of *STEC* cross-transmission between cattle and camels. Furthermore, the hygienic conditions of the camels’ housing and milking conditions were pursued by the owners.

*E. coli* is a Gram-negative rod, representing an important component of the microbiota of mammals and birds. However, several strains of *E. coli*, mainly diarrheagenic *E. coli*, are pathogenic to human and animals and cause several gastrointestinal disorders, including diarrhea [36]. Despite the seriousness of diarrheagenic *E. coli*, especially *STEC*, the studies conducted in Egypt were limited to cattle and sheep [37,38,39], compared with those conducted on camels. Therefore, the milk samples collected from clinically and sub-clinically mastitic she-camels and feces were further examined for the presence of *E. coli*. The results showed that *E. coli* was isolated using conventional culture methods from 25.6 and 19.7% of the examined clinically and sub-clinically mastitic camel’s milk. These findings are lower than those previously obtained by Abo Hashem et al. [40], who reported that *E. coli* was the most predominant isolated bacteria from she-camel’s milk with isolation rates of 35.4 and 27% from apparently healthy and mastitic she-camel’s milk, respectively. Concerning the isolation of *E. coli* from the fecal samples, our results showed that *E. coli* was isolated from 44.4 and 12.3% of the examined fecal samples collected from diarrheic camels and apparently healthy she-camels, correspondingly, as shown in Table 3. Similar detection rates of *E. coli* from she-camels were observed by Al Humam [41], who detected isolates in 26% of cases. Contrariwise, these findings were lower when compared with those formerly reported by El-Hewairy et al. [42] and Al-Ajmi et al. [43]. Conversely, our findings are higher than those reported by Shahein et al. [7], where *E. coli* was isolated from 17.1% of the examined fecal samples collected from diarrheic camels. Several studies were undertaken to assess the prevalence of *E. coli* in fecal samples collected from diarrheic camels in Qatar [44], United Arab Emirates [43], Kenya [45], and Nigeria at 3.8% [46]. However, El-Sayed et al. [18] failed to detect any *STEC* from camel feces. These differences in findings could be attributed to the area of samples collections and the hygienic conditions of the housing and the milking procedures.

Regarding the human stool samples (Table 4), our results showed that *E. coli* was isolated from 23.2 and 37.2% of the examined stool samples collected from contact and non-contact individuals, respectively. Among these isolates, STEC represented 16.7 and 34.5%, respectively, with no statistically significant difference. These findings are similar to those reported by EL-Alfy et al. [47], where *E. coli* was isolated from 31.4% of the examined diarrheic human stools. On the contrary, Ramadan et al. [48] stated that *E. coli* was isolated from 58.6 and 71.4% of the examined diarrheic and healthy individuals’ fecal samples, respectively.

Concerning the seasonal prevalence of STEC, our results revealed a higher prevalence of *STEC* in a cold climate than in hot climates. A similar result was reported by Persson et al. [49], who declared that the prevalence of STEC was more prevalent in the wet season. These findings are inconsistent with those of Monaghan et al. [50] and Moses et al. [51], who reported an increased prevalence of STEC in the summer–early autumn among the camel population. These differences could be attributed to the management process and the isolation techniques used in different laboratories. However, further investigations are required to declare the effect of seasons on the prevalence of STEC.

Our results showed that six different STEC serotypes were recovered from camel samples, including O26, O45, O103, O111, O121, and O145 in percentages of 6, 2, 2, 6, 4 and 6%, respectively, whereas the O26, O45, O103, and O145 serotypes were recovered from human stool samples in percentages of 11.4, 8.6, 5.7, and 5.7%, respectively. These results are nearly comparable to those obtained by Shahein et al. [7], who isolated several *E. coli* serotypes from fecal samples of camel neonates, including O26, O103, O111, and O45 in a percentage of 33.3, 25, 25, and 16.7%, respectively, and Bakhtiari et al. [52], who concluded that the most recovered *STEC* serotypes from human isolates were O26, O45, O103, O111, O121, and O145.

*STEC* represents a significant health problem worldwide as it is accountable for an estimated 2,801,000 acute illnesses yearly [17]. STEC causes many infections in humans, including gastrointestinal illnesses including non-bloody or bloody diarrhea, hemorrhagic colitis, and hemolytic uremic syndrome [53], which has been infrequently identified in camels. The transmission of STEC usually occurs through contaminated foods, water, and person-to-person spread [54,55].

Our results showed that 13 (26%) and 11 (31.4%) of the examined *E. coli* isolates recovered from camel and human samples, respectively, were positive for at least one of the examined genes for STEC. Among the tested camel isolates, the most prevalent virulence factors were *stx*_1_, *eae*, and *stx*_2_ by rates of 46.2, 30.7, and 23.1%, respectively. These results were similar to the finding of Ranjbar et al. [56] and Rashid et al. [57], who stated that *stx*_1_ was the most common virulence gene of STEC, and Mashak [58], who stated that the presence of this large combination of virulence factors increases the pathogenicity of the isolates. In contrast, none of the tested camel isolates were found to have the *hlyA* encoding gene. Despite this, Adamu et al. [46] found that virulence genes were present in substantial amounts in camel STEC and that *stx*_1_ and *stx*_2_ were present in 43.5% of the tested isolates. In addition, the *hlyA* gene was present in 69.6% of the isolates. On the other side, *stx*_2_ was shown to be the most frequently detected in human isolates (45.5%), which is consistent with the findings of Miara et al. [31], Hakim et al. [39], and Adamu et al. [46].

The extensive use of antibiotics in treating infectious diseases and as feed additives has resulted in the emergence of multi-drug-resistant bacteria [59,60,61,62]. The emergence of multi-drug-resistant STEC is one of the concerns of health and food safety authorities worldwide [63,64], as the resistance genes can be reproduced and transmitted not only to other bacteria but also to other hosts, including humans. Previous reports showed that antibiograms are considered more reliable for detecting antibiotic resistance than genotypic resistance gene detection [65]. In the present study, 13 and 11 *STEC* isolates were recovered from camel and human samples, which were screened for antimicrobial susceptibility. The current study results showed that the camel *STEC* isolates were sensitive to the most tested antibiotics, except Oxytetracycline, to which the isolates showed resistance with 53.8%, while the human isolates of STEC showed the highest resistance to Amoxicillin, Gentamycin, and Clindamycin, with ratios of 81.8, 72.7, and 63.6, respectively. The closest results to this study for the resistant strains of several human isolates were reported by Momtaz et al. [66], who determined that the isolates were more resistant to Oxytetracycline 86%, and Ranjbar et al. [67], Gentamycin, Ciprofloxacin, and aminoglycosides. Higher results for antibiotic resistance were observed by Ababu et al. [68], who noted that the resistance of the isolates for both Oxytetracycline and Gentamycin was 100%. On the other hand, Al-Ajmi et al. [43] stated that 100% of STEC isolates were susceptible to Ciprofloxacin and 84% for Amoxicillin. The relatively little discovery of multiple drug-resistant human isolates to many antibiotics in this study may be due to the dependence of people in this region on traditional methods, which may have a prominent effect on maintaining human health [32,33].

Small proportions of the resistance of camel isolates to many antibiotics may be due to the nature of their breeding in these semi-arid desert areas and the lack of excessive use of antibiotics, whether in treatment or as growth stimulants, except for Oxytetracycline [58,66]. A high resistance to Oxytetracycline among camel isolates in our study was discovered, possibly because of the extensive use of these broad-spectrum antibiotics by paramedical personnel and camel holders. None of the isolates were resistant to Clindamycin, which is not surprising because there is no trade medicine for veterinary use that contains Clindamycin for large animal treatment as an active ingredient in Egypt.

## 4. Materials and Methods

### 4.1. Study Area and Animals

This study was conducted in Wadi El-Natroun, which is a semi-arid area in El-Behira governorate, Egypt, located in the Western desert, which is located 23 m (75 ft) below sea level and 38 m (125 ft) below the Nile River level. This study was conducted on 180 she-camels and humans in contact with these animals in the same area.

### 4.2. Sampling

Milk samples: Between 2020 and 2021, 720 milk samples were collected from 180 she-camels (4 udder quarters per animal). The camels were randomly selected, as they are bred sporadically in this semi-arid nomadic region and feed mainly on the grasses that grow in it. A part of each milk sample was tested using the California mastitis test (CMT), and the other portion of milk samples were placed directly in the icebox and sent to the laboratory with minimum delay. The CMT test is used to determine whether or not mastitis is present. Differentiation between subclinical and clinical mastitis was based on the apparent symptoms of mastitis (e.g., swelling and redness of the udder in addition to milk clotting).

Fecal samples: A total of 180 fecal samples were collected from the examined she-camels using rectal swabs in order to reduce potential environmental contamination, then placed directly in an icebox and sent to the laboratory as soon as possible.

Stool samples: A total of 104 stool samples were collected from people living in the same breeding areas as these camels. All the people in this work live in the same study area, some of them are in direct contact with the tested camels, and they carry out various care operations such as milking, providing them with food, and cleaning, and their number is 26. Others live in the same breeding areas only, but they are not in direct contact with the camels, and their number is 78. Then, the samples were placed directly in an icebox and sent to the laboratory with minimum delay. CMT was performed according to the procedure of Hoque et al. [69].

### 4.3. Isolation and Identification of E. coli

Of the tested milk samples, 25 mL was added to 225 mL of buffered peptone water. Additionally, the fecal and stool swabs were immersed in Macconkey broth. The samples were incubated aerobically at 37 °C/24 h. After that, the samples were streaked aerobically on Macconkey agar media at 37 °C/24 h; the suspected colonies were picked up and re-streaked on EMB at 37 °C/24 h for further processing purification. The presumptive green sheen metallic colonies were biochemically tested according to Quinn et al. [70]. The suspected lactose fermenter colonies were first picked up from Macconkey agar media then re-cultured on EMB for further purification. Finally, a pure separate colony was picked up for further investigation and identification.

### 4.4. Serotyping

Serological identification of E. coli isolates was performed according to Kok et al. [71].

### 4.5. Procedures for Determination of O-Antigen Group

Two separate drops of saline were put on a glass slide, and a portion of the colony from the suspected culture was emulsified with the saline solution to give a smooth, reasonably dense suspension. To one suspension, control, one loopful of saline was added and mixed. One loopful of the undiluted antiserum was added to the other suspension and tilted back and forward for one minute. Agglutination was observed using indirect lighting over a dark background. When a colony gave a strongly positive agglutination with one of the pools of polyvalent serum, a different portion of it was inoculated onto a nutrient agar slant and incubated at 37°C for 24 h to grow as a culture for testing with monovalent sera. A heavy suspension of bacteria from each slope culture was prepared in saline, and slide agglutination tests were performed with the diagnostic sera to identify the O-antigen.

### 4.6. PCR Template Preparation

One or two colonies of each confirmed *STEC* isolate were thoroughly mixed in 1 mL of distilled water then boiled for 10 min. The boiled suspension was centrifuged at 1200 rpm/3 min, then 1 μL of supernatant was used as a DNA template.

PCR procedure was carried out in a total volume of 20 μL. Each 20-milliliter PCR reaction mixture contained 10 mL of the 2X Fast Cycling PCR master mix (Qiagen Fast Cycling PCR Kit, Qiagen, Valencia, CA, USA); 4 mL of the primer master mix (*stx*_1_, *stx*_2_, *eae*, and *hlyA*) (Table 9); 5 mL of DNase, RNase-free water; and 1 ml of template DNA (200 e900 ng/mL). The reaction was performed in an Applied Biosystems 2720 thermal cycler under the following conditions.

Samples were subjected to 35 PCR cycles, each consisting of 1 min of denaturation at 95 °C; 2 min of annealing at 65 °C for the first 10 cycles, decrementing to 60 °C by cycle 15; and 1.5 min of elongation at 72 °C, incrementing to 2.5 min from cycles 25 to 35. Amplified DNA fragments were resolved using gel electrophoresis.

### 4.7. Antibiotic Susceptibility

STEC isolates were tested against the different antibiotics according to the CLSI breakpoint [34]. We limited the design of the experiment to the group of antibiotics used in the place of the study, knowing that the Bedouin nature makes them more inclined to use medicinal herbs in treatment. The tested antibiotics were Streptomycin (10 μg/disk), Gentamycin (10 μg/disk), Clindamycin (2 μg/disk), Amoxicillin (30 μg), Ampicillin (10 μg/disk), Oxytetracycline (30 μg), and Ciprofloxacin (5 μg/disk) (Table 10).

### 4.8. Statistical Analysis

The chi-square test was employed to compare differences between different values. A *p*-value of <0.05 was considered to indicate statistically significant differences.

## Figures and Tables

**Table 1:** Occurrence of clinical and subclinical mastitis in she-camel.

**Milk**	180 she-camels	Total (34/180) = 18.9%
		Clinical mastitis 15 (8.3%)
		Subclinical 19 (10.6%)
	720 milk samples per quarter level (180 animals ∗ 4 quarters)	Clinical mastitis 43 (5.9%)
		subclinical 71 (9.9%)
**Fecal Samples**	180	Diarrhea 9 (5%)
		Normal 171 (95%)

**Table 2:** Occurrence of *E. coli* in mastitic she-camel’s milk in relation to camel breeding system (mixed with other species).

**Types of Mastitis**	**Separate (no 72)**		**Mixed Breeding (no 108)**		**Chi-Square Value**	***p***-**Value**
	No.	%	No.	%		
Clinical mastitis	3	4.1	12	11.1	6.58	0.04
Subclinical mastitis	4	5.6	15	13.9		
Total	7	9.7	27	25		

*p*-value is significant at <0.05.

**Table 3:** Isolation of *E. coli* from milk samples/quarter and fecal samples of the examined she-camels.

Camel Samples	*E. coli* Conventional Isolation		STEC (PCR)/Total Cases		STEC (PCR)/*E. coli* Isolates	
	No.	%	No.	%	No.	%
Clinical mastitis/quarter *n* = 43	11	25.6	3	6.9	3	27.3
Subclinical/quarter *n*= 71	14	19.7	6	8.5	6	42.9
Total/quarter *n* = 114	25	21.9	9	7.9	9	36
Diarrhea *n* = 9	4	44.4	1	11.1	1	25
Normal feces *n* = 171	21	12.3	3	1.8	3	14.3
Total *n* = 180	25	13.9	4	2.2	4	16

**Table 4:** Isolation of *E. coli* from human stool samples.

Human Samples	*E. coli* Conventional Isolation		STEC (PCR)/Total Cases		STEC (PCR)/*E. coli* Isolates	
	No.	%	No.	%	No.	%
Contact *n* = 26	6	23.2	1	3.8	1	16.7
Non-contact *n* = 78	29	37.2	10	12.8	10	34.5
Total *n* = 104	35	33.7	11	10.6	11	31.4
Chi-square value	1.73		1.66		1.66	
*p*-value	0.19		0.18		0.18	

**Table 5:** Occurrence of STEC in relation to the hot and cold season.

	Hot Climate	Cold Climate
Clinical mastitis	0	3
subclinical	1	5
Diarrhea	0	1
Normal feces	0	0
Human isolates	1	10

**Table 6:** Serotyping of *E. coli* isolates.

Species	Serotypes of STEC					
	O26 (%)	O45 (%)	O103 (%)	O121 (%)	O145 (%)	O111 (%)
Camel isolates *n*= 13	3 (6)	1 (2)	1 (2)	2 (4)	3 (6)	3 (6)
Human isolates *n* = 11	4 (11.4)	3 (8.6)	2 (5.7)	0	2 (5.7)	0
Total *n* = 24	7	4	3	2	5	3

**Table 7:** Occurrence of virulence factors in relation to isolates.

Species		Virulence Genes							
		*stx*_1_	*stx*_2_	*stx*_1_ & *stx*_2_	*eae*	*hlyA*	*stx*_1_ + *eae*	*stx*_2_ + *eae*	*stx*_1_ & *stx*_2_ + *eae*
STEC Camel isolates (13)	No.	6	3	4	4	0	3	1	0
	%	46.2	23.1	30.7	30.7	0	23.1	7.7	0
STEC human isolates (11)	No.	4	5	1	5	3	1	2	1
	%	36	45.5	9	45.5	27.3	9	18.1	9

**Table 8:** Antibiotic sensitivity test for *STEC* isolates against different antibiotics using CLSI breakpoint [34].

Antibacterial Agent	No. of Resistance among Camel Isolates				No. of Resistance among Human Isolates			
	No.	R (%)	I (%)	S (%)	No.	R (%)	I (%)	S (%)
Streptomycin	13	1(7.7)	2(15.4)	10(77)	11	5 (45.5)	5(45.5)	1(9)
Gentamycin		1(7.7)	4(30.8)	8(61.5)		8 (72.7)	1(9)	2(18.2)
Clindamycin		0(0)	1(7.7)	12(92.3)		7 (63.6)	2(18.2)	2(18.2)
Amoxicillin		0(0)	2(15.4)	11(84.6)		9 (81.8)	1(9)	1(9)
Ampicillin		1(7.7)	3(23.1)	9(69.2)		4 (36.4)	1(9)	6(54.5)
Oxytetracycline		7(53.8)	4(30.8)	2(15.4)		2 (18.2)	4(36.4)	5(45.5)
Ciprofloxacin		1(7.7)	3(23.1)	9(69.2)		7 (63.6)	1(9)	3(27.3)

R = resistant; I = intermediate; S = susceptible.

**Table 9:** Primers used were supplied from Metabion (Germany).

**Target Gene**	**Primer Sequence (5′–3′)**	**Fragment Size (bp)**	[72]
*stx*_1_	F: ATAAATCGCCATTCGTTGACTAC R: AGAACGCCCACTGAGATCATC	180	
*stx*_2_	F: GGCACTGTCTGAAACTGCTCC R: TCGCCAGTTATCTGACATTCTG	255	
*eae*	F: GACCCGGCACAAGCATAAGC R: CCACCTGCAGCAACAAGAGG	384	
*hly* *A*	F: GCATCACAAGCGTACGTTCC R: AATGAGCCAAGCTGGTTAAGCT	534	

**Table 10:** Interpretation criteria.

Antimicrobial Agent	Disk Content	Zone Diameter Interpretive Criteria (Nearest Whole mm)		
Clindamycin	2 μg	S	I	R
Ampicillin	10 μg	≥19	16–18	≤15
Gentamycin	10 μg	≥17	14–16	≤13
Streptomycin	10 μg	≥15	13–14	≤12
Tetracycline	30 μg	≥15	12–14	≤11
Ciprofloxacin	5 μg	≥15	12–14	≤11
Amoxycillin	30 μg	≥31	21–30	≤20

R = resistant; I = intermediate; S = susceptible.

## References

[1] Hussein J., Schuberth H.J. (2020). Recent advances in camel immunology. Front. Immunol..

[2] Bekele T., Zeleke M., Baars R. (2002). Milk production performance of the one-humped camel (*Camelus dromedarius*) under pastoral management in semi-arid eastern Ethiopia. Livest. Prod. Sci..

[3] Yagil R. (2019). Cosmeceuticals: Camel and other milk—Natural skin maintenance. Complementary and Alternative Medicine: Breakthroughs in Research and Practice.

[4] Bashir M.E.A.A. (2014). Studies on Clinical, Aetiological and Antibiotic Susceptibility of Mastitis in She-Camel Camelus dromedarius in Butane Area, Sudan. Ph.D. Thesis.

[5] Husein A., Haftu B., Hunde A., Tesfaye A. (2013). Prevalence of camel (*Camelus dromedaries*) mastitis in jijiga town, Ethiopia. Afr. J. Agric. Res..

[6] Jilo K., Galgalo W., Mata W. (2017). Camel mastitis: A review. MOJ Eco Environ. Sci..

[7] Shaheen M.A., Dapgh A.N., Kamel E., Ali S.F., Khairy E.A., Abuelhag H.A., Hakim A.S. (2021). Advanced molecular characterization of enteropathogenic *Escherichia coli* isolated from diarrheic camel neonates in Egypt. Vet. World.

[8] Abdulkadhim M. (2012). Prevalence of methicillin resistance staphylococcus aureus in cattle and she-camels milk at Al-Qadisyia province. Al-Anbar J. Vet. Sci..

[9] Abeer A., Gouda A., Dardir H., Ibrahim A. (2012). Prevalence of some milk-borne bacterial pathogens threatening camel milk consumers in Egypt. Global Vet.

[10] Saleh S.K., Al-Ramadhan G., Faye B. (2013). Monitoring of monthly SCC in she-camel in relation to milking practice, udder status and microbiological contamination of milk. Emir. J. Food Agric..

[11] Bessalah S., Fairbrother J.M., Salhi I., Vanier G., Khorchani T., Seddik M.M., Hammadi M. (2016). Antimicrobial resistance and molecular characterization of virulence genes, phylogenetic groups of *Escherichia coli* isolated from diarrheic and healthy camel-calves in Tunisia. Comp. Immunol. Microbiol. Infect. Dis..

[12] Alhendi A.B. (2000). Common diseases of camels (*Camelus dromedari*) in eastern province of Saudi Arabia. Pak. Vet. J..

[13] Carvalho I., Tejedor-Junco M.T., Gonzalez-Martin M., Corbera J.A., Silva V., Igrejas G., Torres C., Poeta P. (2020). *Escherichia coli* producing extended-spectrum beta-lactamases (es) from domestic camels in the canary islands: A one health approach. Animals.

[14] Gyles C.L. (2007). Shiga toxin-producing *Escherichia coli*: An overview. J. Anim. Sci..

[15] Kaper J.B., Karmali M.A. (2008). The continuing evolution of a bacterial pathogen. Proc. Natl. Acad. Sci. USA.

[16] Kaddu-Mulindwa D., Aisu T., Gleier K., Zimmermann S., Beutin L. (2001). Occurrence of Shiga toxin-producing *Escherichia coli* in fecal samples from children with diarrhea and from healthy zebu cattle in Uganda. Int. J. Food Microbiol..

[17] FAO (2019). Shiga Toxin-Producing Escherichia coli (Stec) and Food: Attribution, Characterization and Monitoring.

[18] El-Sayed A., Ahmed S., Awad W. (2008). Do camels (*Camelus dromedarius*) play an epidemiological role in the spread of Shiga toxin-producing *Escherichia coli* (stec) infection?. Trop. Anim. Health Prod..

[19] Moore J., McCalmont M., Xu J., Nation G., Tinson A., Crothers L., Harron D. (2002). Prevalence of faecal pathogens in calves of racing camels (*Camelus dromedarius*) in the United Arab emirates. Trop. Anim. Health Prod..

[20] Fadlelmula A., Al-Hamam N.A., Al-Dughaym A.M. (2016). A potential camel reservoir for extended-spectrum beta-lactamase-producing *Escherichia coli* causing human infection in Saudi Arabia. Trop. Anim. Health Prod..

[21] Carvalho I., Silva N., Carroll J., Silva V., Currie C., Igrejas G., Poeta P. (2019). Antibiotic resistance: Immunity-acquired resistance: Evolution of antimicrobial resistance among extended-spectrum β-lactamases and carbapenemases in Klebsiella pneumoniae and *Escherichia coli*. Antibiot. Drug Resist..

[22] Diab M.S., Zaki R.S., Ibrahim N.A., El Hafez M.S.A. (2019). Prevalence of multidrug resistance non-typhoidal salmonellae isolated from layer farms and humans in Egypt. World Vet. J..

[23] Clement M., Olabisi M., David E., Issa M. (2019). Veterinary pharmaceuticals and antimicrobial resistance in developing countries. Veterinary Medicine and Pharmaceuticals.

[24] Erskine R., Walker R., Bolin C., Bartlett P., White D. (2002). Trends in antibacterial susceptibility of mastitis pathogens during a seven-year period. J. Dairy Sci..

[25] Oliver S.P., Murinda S.E., Jayarao B.M. (2011). Impact of antibiotic use in adult dairy cows on antimicrobial resistance of veterinary and human pathogens: A comprehensive review. Foodborne Pathog. Dis..

[26] Massé J., Lardé H., Fairbrother J.M., Roy J.-P., Francoz D., Dufour S., Archambault M. (2021). Prevalence of antimicrobial resistance and characteristics of *Escherichia coli* isolates from fecal and manure pit samples on dairy farms in the province of Québec, Canada. Front. Vet. Sci..

[27] Dego O.K. (2020). Current status of antimicrobial resistance and prospect for new vaccines against major bacterial bovine mastitis pathogens. Animal Reproduction in Veterinary Medicine.

[28] Seligsohn D., Nyman A.K., Younan M., Sake W., Persson Y., Bornstein S., Maichomo M., de Verdier K., Morrell J.M., Chenais E. (2020). Subclinical mastitis in pastoralist dairy camel herds in Isiolo, Kenya: Prevalence, risk factors, and antimicrobial susceptibility. J. Dairy Sci..

[29] Mutua J., Gao C., Bebora L., Mutua F. (2017). Antimicrobial resistance profiles of bacteria isolated from the nasal cavity of camels in Samburu, Nakuru, and Isiolo counties of Kenya. J. Vet. Med..

[30] Suheir I. (2004). Some Bacterial Species, Mycoplasma and Fungal Isolates Associated with Camel Mastitis. Master’s Thesis.

[31] Miara M.D., Bendif H., Ait Hammou M., Teixidor-Tonneau I. (2018). Ethnobotanical survey of medicinal plants used by nomadic peoples in the Algerian steppe. J. Ethnopharmacol..

[32] Owusu E., Ahorlu M.M., Afutu E., Akumwena A., Asare G.A. (2021). Antimicrobial activity of selected medicinal plants from a sub-Saharan African country against bacterial pathogens from post-operative wound infections. Med. Sci..

[33] Aziz M.A., Khan A.H., Adnan M., Ullah H. (2018). Traditional uses of medicinal plants used by indigenous communities for veterinary practices at Bajaur Agency, Pakistan. J. Ethnobiol. Ethnomed..

[34] CLSI (2015). Performance Standards for Antimicrobial Susceptibility Testing; Twenty-Fifth Informational Supplement.

[35] Balemi A., Gumi B., Amenu K., Girma S., Gebru M., Tekle M., Ríus A.A., D’Souza D.H., Agga G.E., Kerro Diego O. (2021). Prevalence of mastitis and antibiotic resistance of bacterial isolates from CMT positive milk samples obtained from dairy cows, camels, and goats in two pastoral districts in southern Ethiopia. Animals.

[36] Alizade H., Hosseini Teshnizi S., Azad M., Shoji S., Gouklani H., Davoodian P., Ghanbarpour R. (2019). An overview of diarrheagenic *Escherichia coli* in Iran: A systematic review and meta-analysis. J. Res. Med. Sci..

[37] Galal H., Hakim A., Dorgham S. (2013). Phenotypic and virulence genes screening of *Escherichia coli* strains isolated from different sources in delta Egypt. Life Sci. J..

[38] Osman K.M., Mustafa A.M., Elhariri M., Abdelhamid G.S. (2013). The distribution of *Escherichia coli* serovars, virulence genes, gene association and combinations and virulence genes encoding serotypes in pathogenic e. Coli recovered from diarrhoeic calves, sheep and goat. Transbound Emerg. Dis..

[39] Hakim A.S., Omara S.T., Same S.M., Fouad E.A. (2017). Serotyping, antibiotic susceptibility, and virulence genes screening of *Escherichia coli* isolates obtained from diarrheic buffalo calves in Egyptian farms. Vet. World.

[40] Abo Hashem M., Ibrahim S., Goda A.S., Enany M. (2020). Diversity of microorganisms associated to she camels’ subclinical and clinical mastitis in South Sinai, Egypt. Suez Canal Vet. Med. J. SCVMJ.

[41] Al Humam N.A. (2016). Special biochemical profiles of *Escherichia coli* strains isolated from humans and camels by the Vitek 2 automated system in al-ahsa, Saudi Arabia. Afr. J. Microbiol. Res..

[42] El-Hewairy H., Awad W., Ibrahim A. (2009). Serotyping and molecular characterization of *Escherichia coli* isolated from diarrheic and in-contact camel calves. Egypt. J. Comp. Pathol. Clin. Pathol..

[43] Al-Ajmi D., Rahman S., Banu S. (2020). Occurrence, virulence genes, and antimicrobial profiles of *Escherichia coli* o157 isolated from ruminants slaughtered in al ain, united Arab emirates. BMC Microbiol..

[44] Mohammed H.O., Stipetic K., Salem A., McDonough P., Chang Y.F., Sultan A. (2015). Risk of *Escherichia coli* o157: H7, non-o157 Shiga toxin-producing *Escherichia coli*, and campylobacter spp. In food animals and their products in Qatar. J. Food Prot..

[45] Baschera M., Cernela N., Stevens M.J.A., Liljander A., Jones J., Corman V.M., Nuesch-Inderbinen M., Stephan R. (2019). Shiga toxin-producing *Escherichia coli* (stec) isolated from fecal samples of African dromedary camels. One Health.

[46] Adamu M.S., Ugochukwu I.C.I., Idoko S.I., Kwabugge Y.A., Abubakar N.S., Ameh J.A. (2018). Virulent gene profile and antibiotic susceptibility pattern of Shiga toxin-producing *Escherichia coli* (stec) from cattle and camels in Maiduguri, north-eastern Nigeria. Trop. Anim. Health Prod..

[47] EL-Alfy S.M., Ahmed S.F., Selim S.A., Aziz M.H.A., Zakaria A.M., Klena J.D. (2013). Prevalence and characterization of Shiga toxin o157 and non-o157 enterohemorrhagic *Escherichia coli* isolated from different sources in Ismailia, Egypt. Afr. J. Microbiol. Res..

[48] Ramadan H., Awad A., Ateya A. (2016). Detection of phenotypes, virulence genes and phylotypes of avian pathogenic and human diarrheagenic *Escherichia coli* in Egypt. J. Infect. Dev. Ctries.

[49] Persson S., Olsen K.E., Scheutz F., Krogfelt K.A., Gerner-Smidt P. (2007). A method for fast and simple detection of major diarrhoeagenic *Escherichia coli* in the routine diagnostic laboratory. Clin. Microbiol. Infect..

[50] Monaghan A., Byrne B., Fanning S., Sweeney T., McDowell D., Bolton D.J. (2011). Serotypes and virulence profiles of non-o157 Shiga toxin-producing *Escherichia coli* isolates from bovine farms. Appl. Environ. Microbiol..

[51] Moses A., Egwu G., Ameh J. (2012). Antimicrobial-resistant pattern of e. Coli o157 isolated from human, cattle and surface water samples in northeast Nigeria. J. Vet. Adv..

[52] Bakhtiari N.M., Fazlara A., Jorge H. (2018). Risk of Shiga toxin-producing *Escherichia coli* infection in humans due to consuming unpasteurized dairy products. Int. J. Enteric. Pathog..

[53] Yamasaki E., Watahiki M., Isobe J., Sata T., Nair G.B., Kurazono H. (2015). Quantitative detection of Shiga toxins directly from stool specimens of patients associated with an outbreak of enterohemorrhagic *Escherichia coli* in Japan—Quantitative Shiga toxin detection from stool during ehec outbreak. Toxins.

[54] Sethulekshmi C., Latha C., Anu C. (2018). Occurrence and Quantification of Shiga Toxin-Producing Escherichia coli from Food Matrices. Vet. World.

[55] Angulo F.J., Steinmuller N., Demma L., Bender J.B., Eidson M., Angulo F.J. (2006). Outbreaks of enteric disease associated with animal contact: Not just a foodborne problem anymore. Clin. Infect. Dis..

[56] Ranjbar R., Masoudimanesh M., Dehkordi F.S., Jonaidi-Jafari N., Rahimi E. (2017). Shiga (Vero)-toxin-producing *Escherichia coli* isolated from the hospital foods; virulence factors, o-serogroups and antimicrobial resistance properties. Antimicrob. Resist. Infect. Control.

[57] Rashid M., Kotwal S.K., Malik M., Singh M. (2013). Prevalence, genetic profile of virulence determinants and multidrug resistance of *Escherichia coli* isolates from foods of animal origin. Vet. World.

[58] Mashak Z. (2018). Virulence genes and phenotypic evaluation of the antibiotic resistance of Vero toxin-producing *Escherichia coli* recovered from milk, meat, and vegetables. Jundishapur J. Microbiol..

[59] Aly S.M., Albutti A. (2014). Antimicrobials use in aquaculture and their public health impact. J. Aquac. Res. Dev..

[60] Shrivastava S.R., Shrivastava P.S., Ramasamy J. (2018). World health organization releases global priority list of antibiotic-resistant bacteria to guide research, discovery, and development of new antibiotics. J. Med. Soc..

[61] Diab M.S., Ibrahim N.A., Elnaker Y.F., Zidan S.A., Saad M.A. (2021). Molecular detection of staphylococcus aureus enterotoxin genes isolated from mastitic milk and humans in el-behira, Egypt. Int. J. One Health.

[62] Abdo S.E., Gewaily M.S., Abo-Al-Ela H.G., Almere R., Soliman A.A., Elkomy A.H., Dawood M.A.O. (2021). Vitamin c rescues inflammation, immunosuppression, and histopathological alterations induced by chlorpyrifos in Nile tilapia. Environ. Sci. Pollut. Res..

[63] Wenz J., Barrington G., Garry F., Ellis R., Magnuson R. (2006). *Escherichia coli* isolates’ serotypes, genotypes, and virulence genes and clinical coliform mastitis severity. J. Dairy Sci..

[64] De Verdier K., Nyman A., Greko C., Bengtsson B. (2012). Antimicrobial resistance and virulence factors in *Escherichia coli* from Swedish dairy calves. Acta Vet. Scand..

[65] Scaria J., Warnick L.D., Kaneene J.B., May K., Teng C.H., Chang Y.F. (2010). Comparison of phenotypic and genotypic antimicrobial profiles in *Escherichia coli* and salmonella enterica from the same dairy cattle farms. Mol. Cell Probes.

[66] Momtaz H., Farzan R., Rahimi E., Safarpoor Dehkordi F., Sound N. (2012). Molecular characterization of Shiga toxin-producing *Escherichia coli* isolated from ruminant and donkey raw milk samples and traditional dairy products in Iran. Sci. World J..

[67] Ranjbar R., Safarpoor Dehkordi F., Sakhaei Shahreza M.H., Rahimi E. (2018). Prevalence, identification of virulence factors, o-serogroups and antibiotic resistance properties of Shiga-toxin-producing *Escherichia coli* strains isolated from raw milk and traditional dairy products. Antimicrob. Resist. Infect. Control.

[68] Ababu A., Endashaw D., Fesseha H. (2020). Isolation and antimicrobial susceptibility profile of *Escherichia coli* o157: H7 from raw milk of dairy cattle in holeta district, central Ethiopia. Int. J. Microbiol..

[69] Hoque M.N., Das Z.C., Talukder A.K., Alam M.S., Rahman A.N. (2015). Different screening tests and milk somatic cell count for the prevalence of subclinical bovine mastitis in Bangladesh. Trop. Anim. Health Prod..

[70] Quinn P.J., Markey B.K., Leonard F.C., Hartigan P., Fanning S., Fitzpatrick E. (2011). Veterinary Microbiology and Microbial Disease.

[71] Kok T., Worswich D., Gowans E., Collee J., Fraser A., Marmion B., Simmons A. (1996). Some serological techniques for microbial and viral infections. Practical Medical Microbiology.

[72] Paton J.C., Paton A.W. (1998). Pathogenesis and diagnosis of Shiga toxin-producing *Escherichia coli* infections. Clin. Microbiol. Rev..

